# Managing the Unpredictable: Recommendations to Improve Trainee Safety During Global Health Away Electives

**DOI:** 10.5334/aogh.3874

**Published:** 2022-10-11

**Authors:** Matthew Edwards, Nisha Dalvie, Anne Kellett, Michael J. Peluso, Robert M. Rohrbaugh

**Affiliations:** 1 Case Western Reserve University School of Medicine, Cleveland, Ohio, USA; 2 Boston Children’s Hospital, Boston, Massachusetts, USA; 3 Yale School of Medicine, New Haven, Connecticut, USA; 4 University of California, San Francisco, San Francisco, California, USA

**Keywords:** Trainee safety, global health, international elective, clinical rotation, pre-departure training

## Abstract

**Background:**

For institutions offering global health programs, the safety of trainees during clinical rotations at international sites is paramount. Current guidelines for global health electives recommend pre-departure training and safety-net resources, yet their advice on managing unanticipated problems is limited.

**Objective:**

This report illustrates critical safety considerations requiring additional guidance for programs and students and highlights approaches that may improve trainee safety while abroad.

**Methods:**

We present a series of five cases adapted from the experiences of students traveling to and from the Yale School of Medicine between the years of 2011–2021. These cases include instances of personal injury, mental health challenges following trauma, sexual harassment, political instability, and natural disaster. For each case, we recommend ways in which programs and their participants may approach the challenges and we highlight issues requiring additional analysis.

**Findings:**

We categorized the types of trainee safety issues into three groups: personal health emergencies, individual-level stressors, and large-scale crises.

**Conclusion:**

Ultimately, we recommend that rather than solely emphasizing a universal policy, programs and trainees should also be educated on the tools and resources available for addressing unexpected emergencies.

## Introduction

The safety of trainees during clinical rotations away from the home institution is a priority for every global health program. Safety guidelines should address preventive measures for the most common safety issues that may arise during an away rotation, both for students traveling from high income countries (HICs) to low-middle income countries (LMICs) and vice versa. Current guidelines for global health electives therefore recommend pre-departure training and safety-net resources such as health and evacuation insurance. For example, since motor vehicle accidents cause the majority of injuries for international travelers to LMICs [1,2], many safety guidelines warn against using motorcycles, public buses, and other forms of road travel while rotating at these sites. Safety guidelines for trainees traveling from LMICs to HICs are less frequently documented in the global health elective literature [3]. General recommendations for all international visitors, including those traveling to HICs, address awareness of automobile-dominated traffic patterns and nuances related to personal safety and privacy, including avoiding alcohol and drug use, never walking alone after sunset, and protecting personal valuables [4]. Additionally, countries with government-based resources, such as the US Safe Traveler Enrollment Program (STEP) and the UK Foreign and Commonwealth Office (FCO), provide tools for citizens and visitors to be aware of travel notifications [5,6].

Although these government-based resources are detailed in their recommendations for addressing well-known safety issues, their advice on managing unanticipated problems is understandably more limited. The need for additional guidance has become apparent in the challenges that students have faced during away experiences over the past ten years of our global health education programs. To illustrate situations in which further guidance may be needed, we present a series of five challenges faced by our trainees and either not anticipated in their pre-departure orientations, or not covered in their orientation at the host site. For each case, we recommend ways in which programs and their participants may approach the problems and we highlight issues requiring additional analysis.

## Methods

The cases presented here were adapted from the experiences of students traveling to or from the Yale School of Medicine during global health clinical elective rotations between the years 2011–2021. The Yale School of Medicine’s Office of Global Health Education (OGHE) supports bilateral exchanges with institutions in eight LMICs. Additionally, Yale’s OGHE receives visiting international medical students from non-affiliated medical schools for short-term clinical electives. For each program, the OGHE provides orientation before departure or upon arrival, covering logistical, cultural, and safety information. In all cases, identifying information was altered to protect the identity of students and partner sites.

## Cases

**Case 1 Personal Injury**

A student from an HIC is completing an elective in Lima, Peru and decides to take a bus trip to the high Andes for a weekend excursion. As the bus rounds a corner in the mountains, it collides with a truck. The student is taken to the nearest local hospital for stabilization of multiple orthopedic injuries, including a spinal fracture. After ten days, doctors at the hospital report that the student is well enough to leave the hospital. The student is still experiencing significant pain and asks a local medical student to send the spinal radiography report to a radiologist at their home institution. The home institution’s radiologist interprets the radiographs to suggest that the student’s cervical spine requires additional stabilizing procedures. The local physicians are upset that the student has sent the radiology reports “behind their back” and adds that these procedures could not be carried out at that hospital anyway due to technical considerations. Consultation with the air-evacuation service contracted by the school reveals that their airplane is unable to operate at the heights required to land in this high-altitude setting.

**Case 2 Mental Health after Trauma**

A student from an MIC is completing a surgery elective in the US. After having spent eight hours in the operating room, the student is walking home after sunset when he is approached by three men who demand that he give them his wallet. When he refuses, one of the individuals pulls out a gun and points it in the student’s face, saying that he will kill the student if he doesn’t give him his wallet. The student hands over the wallet and walks home to the room he is renting, where he is distraught and unable to sleep. He has been looking forward to this elective for over a year and does not want to disrupt it, so he goes into work the next day, where he is distracted, tired, and unable to concentrate. This behavior continues the next day and residents on the team begin to wonder why the student has become so slow and timid. After a week, they talk with the Chief Resident about their concerns. The Chief Resident takes the student aside and asks if something is the matter. The student confides what happened and the Chief Resident attempts to arrange psychological services. However, his travel insurance does not cover mental health treatment and so the Chief Resident is unable to provide these services.

**Case 3 Sexual Harassment**

A medical student from the US is completing a clinical rotation in Brazil and is adjusting to a more openly affectionate culture than that of her home. Although she becomes familiar with the routine of greeting colleagues, and even patients, with a light kiss on the cheek, she still prefers a bit of personal space. Three weeks into her rotation, her home institution reaches out routinely to remind her of post-elective requirements. In doing so, the student takes the opportunity to share a recent experience that left her uncomfortable around one of her assigned physician supervisors. She shares that one physician has routinely been a bit too close in their interactions, which has made her extremely uncomfortable. She has elected not to say anything because she has only a week left in her rotation and doesn’t want to jeopardize the relationship between the two institutions. Her solution to the situation, she shared, was to just avoid being on rotations with the attending. However, she wanted her home institution to be aware of the situation in order to avoid future students experiencing similar discomfort.

**Case 4 Political Instability**

An American medical student is participating in a global health elective in Quito, Ecuador. Two weeks into her rotation, violent protests break out around metro areas in Quito, including fires and large public demonstrations. Government response includes military personnel being posted throughout the city with authorization to use tear gas and water hoses, along with an enforced curfew. The student is instructed by the hospital administration to not attend clinic rotations and remain indoors until further notice. The student establishes phone and email contact with away site administration, home site administration, and the emergency evacuation insurance office to update them daily on her safety and receive updates on pertinent travel information. The local US embassy and the US State Department emergency programs are notified of the student’s situation but offer no resources or other support.

**Case 5 Natural Disaster**

Two visiting medical students, one from the Caribbean and one from South Asia, are halfway through their clinical rotations in the US when the COVID-19 pandemic erupts. As decisions begin circulating to the clinical departments, they each learn that their rotations are being suspended and it is uncertain as to when students would be permitted back on the wards. The announcement is disappointing and raises emotions in the two students as they follow the news in their home countries and wonder whether they will be able to return home.

## Discussion

These cases highlight critical safety considerations that were either not addressed during these students’ pre-departure trainings or not addressed in their in-country elective orientation. While the events outlined in these cases relate the experiences of individuals, we believe that these stories connect with larger, more generalizable concerns. Ensuring trainees and institutions have a better understanding of principles to manage these unpredictable situations is critical to the safety of trainees.

We can categorize the types of trainee safety issues into three groups: personal health emergencies, individual-level stressors, and large-scale (e.g., regional or national level) crises. Personal health emergencies can result from trauma or illness, and involve the logistics and challenges of receiving medical care or evacuation. Individual stressors include events that impact a student’s emotional well-being, such as adjusting to a new culture or experiencing sexual harassment. Large-scale crises encompass events impacting the community in which the student works, including natural disaster, terrorist attack, and civil unrest. Each division presents unique considerations regarding preparation measures and in-situ responses. A summary of recommendations, both previously reported in “gold-standard guidelines” and which we propose here, is provided in Table 1.

**Table 1:** Summary of safety recommendations for global health trainees.


UNANTICIPATED CONCERN	PERSONAL HEALTH EMERGENCY (CASE 1)	EXTERNAL TRAUMA (CASES 2 AND 3)	COUNTRY-WIDE CRISIS (CASES 4 AND 5)

**Prevention**	– Avoid hazardous methods of transportation (e.g., motorcycles). – Understand crime patterns; avoid traveling alone in high crime areas, especially at night. – Establish procedures for accidental exposures, chemical prophylaxis, and PPE requirements. – Link trainees and institutions for choosing health and evacuation insurance that meets individual health and site-specific needs.	– Provide training on away institution sexual harassment reporting and counseling policies as part of pre-departure orientation. – Establish a method of communication and identify a contact person. – Plan administrative/institutional response to handling cases of sexual harassment. – Assign a liaison at the student/administration interface to modify rotation requirements and protect student privacy. – Review insurance to ensure mental health concerns are a valid reason for evacuation. – Screen for trainee mental health concerns during regular check-ins (weekly/biweekly). – Review insurance for coverage of trauma-focused counseling, especially if the away institution does not have similar resources available for trainees.	– Enroll in government-based program (e.g., STEP). – Establish regular communication between the trainee and institutions (weekly or biweekly)

**Resources**	– Ensure access to health, disability, and evacuation insurance while abroad. – Create a contingency fund and advocate for additional resources.	– Ensure access to trauma and sexual harassment-focused counseling from home and/or away institutions; if these resources are insufficient, activate evacuation response. – Establish clear procedures for reporting cases of sexual harassment or assault within the away institution administration.	– Reach out to emergency contacts. – Follow daily communication plan between trainee, home institution, away institution, and necessary external resources; the away institution should provide hyperlocal safety information for all other parties. – Create clear thresholds to activate various levels of emergency response between administrations (i.e., when to suspend rotations, when to evacuate trainees).


### Institutional Responsibility for Insurance

Global health guidelines broadly recommend that trainees obtain evacuation and health insurance, without elaborating on coverage details. As demonstrated in the first two cases, these safety net services may have gaps that leave trainees unable to access physical and mental health care. It is crucial that coverage exceptions be well-understood, and it is unreasonable to expect trainees to navigate complex policies on their own, particularly when insurance needs vary between students depending on their baseline health status and home country insurance coverage. We recommend that purchasing evacuation or health insurance be a shared decision-making process between institutions and their trainees, with individualized health or site-specific concerns addressed prior to travel. We further suggest that institutions designate contingency funds and advocate for additional resources should their trainees require assistance that is not covered by their insurance.

### Advocate Support at Home and Away Institutions

These cases highlight the need for an accessible advocate, at both the home and away institution, whose role includes assisting the trainee in resolving sensitive situations within the context of the need to meet rotation requirements. This role may have prevented the student in Case 2 from needing to divulge personal trauma to a Chief Resident who was also their evaluator, and may have prevented the student in Case 3 from potentially needing to confront her aggressor. In both situations, the power dynamic was skewed against the student and made it difficult to disclose painful events to the supervisor. To ensure that trainees have a safe space to share and process mental trauma, programs should establish a designated point person (either an individual or an office) from whom students can confidentially receive assistance. This role should have the authority to provide support and liaise with the student’s home institution if further services are needed.

### Expanded Emergency Communication Plan

**Figure 1:**
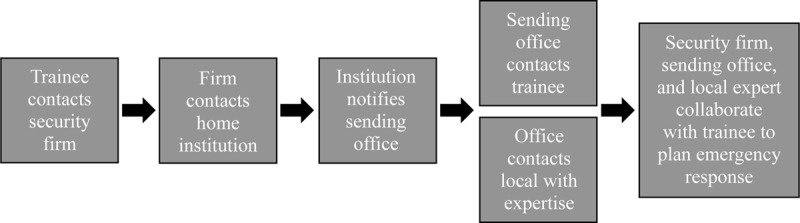
Sample daily communication plan used by our institution during emergency situations.

Nearly all US-based global health guides recommend governmental resources, including the US State Department Travel Advisory, Smart Traveler Enrollment Program (STEP), and US embassies, as a way to keep abreast of current information during crisis situations abroad and potentially obtain immediate assistance. Unfortunately, these resources cannot always provide the most accurate local information, nor can they reliably offer assistance for trainees who need urgent advice, as demonstrated in Case 4. Programs relying on these resources to protect their trainees during national emergencies will likely find them to be insufficient. We recommend that programs establish a communication plan between their trainees, a responsible faculty or staff member both at the away and the home institutions, and third-party security firms that specialize in international health and risk management. These communication plans must go beyond the emergency contact cards recommended in the current global health guides; an example of the level of detail required to maximize safety is illustrated in Figure 1.

Regardless of what information sources the home institution uses to monitor global crises, the away institution must be able to provide localized updates tailored to the trainee’s location, as this information will be more useful than higher-level travel risk assessments provided by the government or media. During crises, check-ins between all parties should increase to at least daily, especially in rapidly evolving situations. These check-ins would provide trainees the opportunity to voice their mental and physical safety concerns and to ensure that there is agreement on the situation’s urgency. Personal updates relayed by the trainees, on-the-ground information from away sites, and institutional recommendations should all be taken into account when developing individualized emergency plans, a specialty of third-party security entities.

### Negotiating a Threshold

Global health guidelines define an “emergency situation” as personal health emergencies, natural disasters, and other scenarios that prevent trainees from safely completing their rotation. The above cases clearly fit this definition in hindsight, yet it is often unclear in real-time whether trainees should initiate emergency protocols. This is demonstrated in Case 5: first, before the number of COVID-19 cases had reached the point of threatening trainee safety, and second, when the emergency plan of returning to the home institution was also unsafe. Although the situation was unprecedented, there was no clear threshold provided by either the home or the away institution concerning how and at what point the trainees should be evacuated. In Case 3, the trainee herself was unclear whether her situation qualified as an emergency that required additional resources, leading to the harassment not being addressed during her rotation. This lack of clarity for a threshold can also be attributed to cultural competency frameworks that emphasize yielding to local customs and yet do not clearly define a point at which a trainee’s discomfort may indicate that their safety is compromised. We therefore recommend that programs and their trainees negotiate the threshold that defines an emergency and identify which resources are available for support. We suggest that the emergency threshold include not only events that threaten a trainee’s physical safety, but also their learning climate.

### Personal Comfort versus Cultural Norms

Students traveling to away rotations may enter environments with radically different workplace and cultural norms than their own. Some of these norms may challenge or violate the student’s expectations for a professional and safe working environment. Navigating instances of workplace sexual harassment is universally complex and is made more so in an unfamiliar setting. Differences in cultural norms may create challenges to addressing sexual harassment, as trainees may be unaware of which behaviors violate local norms and may be unfamiliar with the differences in workplace policies between home and away institutions.

The complexity of addressing sexual harassment in non-homogenous cultural and institutional settings makes a universal policy unrealistic. Rather, trainees, home institutions, and away institutions should prioritize the discussion of cultural norms so that all participants arrive at agreed-upon expectations for professional behavior. Trainees should receive pre-departure training on away institutional sexual harassment reporting policies, home and/or away institution resources for sexual harassment victim counseling, contacts within the home institution for accessing additional resources, and orientation at the host site, which is best equipped to provide information on policies, local contacts, and customs. Trainees should establish a method of communication with their home institution and a designated point of contact to report situations of sexual harassment. The point of contact should have access to resources from the home and away institution to support the trainee and escalate the concern if necessary.

### Longitudinal Student Support

“Post-return debrief” is a well-known concept emphasized in the global health education literature [7,8]. However, this debrief should not be considered the only opportunity for students to process trauma beyond typical culture shock, as experienced by the trainees in Case 2 and Case 3. Emergency preparation entails both home and away institutions being able to connect students with mental health resources regardless of timing. Examples of such resources include telehealth visits with therapy in the home country, in-person therapy with counselors covered by travel health insurance, and other counseling opportunities provided by the away site. Importantly, students should be familiar with these options prior to departure. The need for these additional resources should be screened for by home or away site staff as part of regularly scheduled check-ins during the rotation, per the schedule recommended by current global health guidelines, rather than occurring at the end of the rotation. For concerns regarding sexual harassment, these resources and check-ins should also review how the trainee’s away site handles sexual harassment incidents and the home institution’s policy for addressing conflicts with the other institution’s policies.

### Limitations of this Case Series

By our own argument, this report cannot encompass all scenarios that may arise to threaten the safety of global health trainees. The types of challenges imposed by international experiences are limitless and constantly evolving. Rather than creating a single set of guidelines that accounts for all individual, institutional, and site differences, a program should elicit, value, and incorporate the feedback of its student participants, particularly for less common scenarios that are not covered by current institutional protocols. Future studies will benefit from a wider sampling of trainees, using open-ended interviews to find common themes between institutions.

## Conclusion

The unscripted nature of international rotations is at the core of global health education, as these experiences promote critical reflection and transformative learning. Unfortunately, however, these same experiences can also compromise trainee safety. Programs and students must be as aware of resources for managing emergencies as for more common threats. The “gold standard” guidelines provide excellent coverage of basic safety principles and how to minimize risk of personal injury and disease. Managing these situations is emphasized within the literature, often in the form of pre-travel checklists for programs and trainees. The unpredictable nature of emergencies demands an equally robust system for identifying and managing the fringe cases that are not addressed by these checklists. More importantly than creating a universal policy, trainees and programs should be deliberately educated on the tools and resources available for addressing unexpected emergencies. Through this case series, we hope to enable individuals and institutions to establish a more complete approach to safety that supports trainees of both HICs and LMICs throughout their global health experiences.
